# Epidemiologic and Molecular Investigation of a MRSA Outbreak Caused by a Contaminated Bathtub for Carbon Dioxide Hydrotherapy and Review of the Literature

**DOI:** 10.1155/2020/1613903

**Published:** 2020-04-14

**Authors:** Claas Baier, Ella Ebadi, Tobias R. Mett, Matthias Stoll, Gerald Küther, Peter Maria Vogt, Franz-Christoph Bange

**Affiliations:** ^1^Institute for Medical Microbiology and Hospital Epidemiology, Hannover Medical School, Hannover, Germany; ^2^Department of Plastic, Aesthetic, Hand and Reconstructive Surgery, Burn Center, Hannover Medical School, Hannover, Germany; ^3^Clinic for Immunology and Rheumatology, Hannover Medical School, Hannover, Germany; ^4^Department of Rehabilitation Medicine, Hannover Medical School, Hannover, Germany

## Abstract

**Methods:**

We conducted an outbreak investigation and performed a molecular typing of the outbreak strains with pulsed-field gel electrophoresis (PFGE). In addition, we reviewed PubMed and the Outbreak Database for MRSA outbreaks related to hydrotherapy or other bathing activities.

**Results:**

Four patients acquired nosocomial MRSA during the 4-week outbreak period. Environmental sampling revealed the presence of MRSA in the bathtub used for hydrotherapy. The environmental and the patients' isolates showed an indistinguishable restriction pattern in the PFGE. Subsequent discontinuation of bathing stopped the outbreak. The literature search found 9 MRSA outbreak reports related to bathing activities or hydrotherapy.

**Conclusion:**

The epidemiologic outbreak investigation together with the molecular findings suggests monoclonal spread of MRSA due to surface contamination of the bathtub. After enhancing the disinfection and cleaning process accompanied by staff training with respect to hand hygiene, no further cases occurred. Standardized and best practice cleaning and disinfection protocols are crucial, especially in critical facilities such as hydrotherapy units. Regular environmental sampling is helpful to monitor these processes and to detect potential contamination.

## 1. Introduction

Immersion in a carbon dioxide-enriched water bath is a traditional therapeutic approach in patients with skin and soft tissue diseases [1]. Topical carbon dioxide application can improve the skin microcirculation [2] and may help skin regeneration [3]. Depending on the extent of the underlying lesions, only a body part can be treated or the entire patient may be exposed to the carbon dioxide. During this treatment, bacteria from the patient's microbiome (including when present multidrug resistant bacteria such as methicillin-resistant *Staphylococcus aureus*; MRSA) can contaminate the bathtub and its surroundings [4]. MRSA is also found in bathtubs in normal household settings [5]. In the literature, there are some reports addressing contaminated hydrotherapy equipment as a (point) source of nosocomial spread of bacteria [6, 7].

Here we describe the characteristics and control of a MRSA outbreak which affected 4 adult patients from different wards in our clinic recognizing the ORION statement [8]. In addition, we provide an overview concerning MRSA outbreaks associated with hydrotherapy and other bathing activities.

## 2. Materials and Methods

### 2.1. Setting and Basic Infection Control Measures

The hydrotherapy unit is located in the Department of Rehabilitation Medicine offering carbon dioxide therapy for patients of all specialties. It provides two full-size bathtubs for carbon dioxide treatment. One tub is equipped with a patient lift to transport immobile or obese patients.

After each usage, the bathtubs were emptied, cleaned, and then disinfected by dedicated hydrotherapy staff. For wipe disinfection of the bathtubs, a commercially available bactericidal product with phenoxyethanol, N,N-bis-(3-Aminopropyl)dodecylamine, and benzalkonium chloride (Incidin™ Pro 0,5%, ECOLAB Healthcare) was used. In addition, premoistened wipes containing ethanol and isopropyl alcohol (mikrozid® universal wipes premium maxi, Schülke) were available for disinfection of smaller surfaces. Patients carrying multidrug-resistant bacteria (colonized or infected) were allowed to use the bathtubs. Carbon dioxide-enriched water was mixed with tap water to fill the tub. Patients undergoing carbon dioxide therapy were supervised by staff members during the procedure. The therapists wore personal protective equipment (PPE; i.e., gown and gloves) during bathing. Disinfectant dispensers for alcoholic hand hygiene were located directly near the tubs.

### 2.2. Outbreak Investigation

We reviewed and collected movement data and microbiology results of patients who received carbon dioxide treatment in the hydrotherapy unit during the outbreak period. All patients that acquired nosocomial MRSA (i.e., detection of MRSA on day 3 or later of the hospital stay without a prior history of MRSA) with the same phenotypic antimicrobial susceptibility pattern were presumed outbreak cases. A pulsed-field gel electrophoresis was performed according to an in-house protocol with *SmaI* as the restriction enzyme and electrophoresis in 1% agarose gel in order to examine molecular relationship of the strains. The band patterns were visually compared and evaluated based on the criteria described by Tenover et al. [9]. In addition, the spa-type of the outbreak isolates was determined. Microbiology specimens were cultured using standard methods. Antibiotic susceptibility testing of the isolates was performed with the VITEK 2 system (bioMérieux, Marcy-l'Étoile, France). Environmental specimens were collected at different locations (tub surface at several positions, sink, showerhead, water tap, patient lifter, neck roll, disinfectant container, and disinfectant plastic packaging) with contact plates (Tryptone Soya Agar with neutralizers tween, lecithin, and histidine; Oxoid, PO5024C, Wesel, Germany) and swabs.

### 2.3. Literature Overview

We performed a literature search to identify reports that link MRSA outbreaks with the use of hydrotherapy or other bathing activities. The search was performed in PubMed (http://www.pubmed.gov) using the following algorithms: (1) MRSA and pool; (2) MRSA and hydrotherapy; (3) MRSA and bathtub; (4) hydrotherapy and outbreak; and (5) bathing and outbreak. The articles found were restricted to reports published in English between 1980 and 2018. In addition, we searched the Outbreak Database (http://www.outbreak-database.com).

## 3. Results

### 3.1. Epidemiologic Outbreak Characteristics

Twenty-four patients were treated in the 4 week long outbreak period in the bathtub with the patient lifter. Overall, 4 of these (cases 1 to 4, all adults) acquired nosocomial MRSA (Table 1). As the phenotypic antimicrobial susceptibility patterns of the MRSA strains were identical, these 4 patients were considered as outbreak cases. The first 3 patients acquired MRSA within the same week (week one of the outbreak). MRSA acquisition took place within two weeks after each patient had begun the carbon dioxide therapy. Two of the patients came from the same surgical ward; the third patient was located on the tertiary burn unit, and the fourth patient was on an internal medicine ward at the time point of MRSA detection. The third case triggered the outbreak investigation. Nosocomial MRSA acquisition on the burn unit became easily apparent as the ward has only 6 patient beds and a tight microbiological monitoring. The analysis of movements and treatment data showed that the first 3 patients were repeatedly treated in the same bathtub immediately prior to their first MRSA findings. On one occasion, all 3 patients were treated at the same day in this tub. This finding raised the suspicion of a cross-transmission in the bathtub so that an environmental sampling was performed. On the day before the environmental examination revealed growth of MRSA, a fourth patient who had repeatedly undergone carbon dioxide treatment in the same tub acquired MRSA (week 4) as well. This patient tested positive for MRSA about 3 to 4 weeks after the beginning of carbon dioxide bathing. Case 4 and case 2 were treated at the same day in the bathtub on cumulative 6 days prior to the first MRSA finding in case 4. Case 2 was already positive for MRSA during this period.

### 3.2. Microbiological and Molecular Typing Findings

All patients had negative specimens for MRSA prior to the bathing therapy: cases 1 to 3 had negative screening specimens on admission to the hospital (nasopharyngeal swabs), and all cases had several negative swabs from skin/soft tissue samples. There was no general MRSA screening before the start of hydrotherapy. The hospital-acquired MRSA strains were found in specimens taken from skin and soft tissue in patients 1 to 3. The fourth patient had positive MRSA findings in nasopharyngeal and rectal swabs, and at a further location, that was not specified. All of the patients' MRSA isolates showed an identical antimicrobial susceptibility phenotype including resistance to oxacillin and fluroquinolones while being susceptible to vancomycin, clindamycin, daptomycin, rifampicin, co-trimoxazole, linezolid, and mupirocin. Environmental sampling showed growth of MRSA at 3 different locations (bathtub sink, showerhead, and exterior surface of disinfectant container). The isolates from the showerhead and the disinfectant container showed the same phenotypic resistance profile as the patients' isolates. The strain recovered from the sink, however, was susceptible to fluoroquinolones.

**Figure 1:**
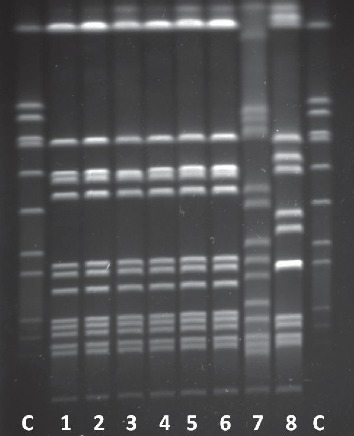
Pulsed-field gel electrophoresis of MRSA isolates. C = *Staphylococcus aureus* control strain NCTC® 8325. 1–4: outbreak cases 1 to 4. 5: showerhead. 6: disinfectant container. 7: sink. 8: MRSA strain of a patient not belonging to the outbreak. The isolates 1 to 6 are monoclonal according to the restriction pattern.

In addition, growth of methicillin-susceptible *Staphylococcus aureus* (bathtub surface and plug) and *Enterobacter* species (disinfectant plastic packaging) was found in three additional environmental samples. The patients' MRSA isolates and two of the MRSA positive environmental samples (showerhead and disinfectant container) showed a monoclonal banding pattern in the PFGE (“indistinguishable” according to Tenover et al. [9]); (Figure 1). Those isolates had the spa-type t020. The MRSA strain from the sink showed a different PFGE pattern in accordance with the differing antibiotic susceptibility pattern.

### 3.3. Outbreak Control

Immediately after the positive environmental sampling became evident, carbon dioxide treatment in both bathtubs was suspended. The infection control staff then performed repeated onsite audits to evaluate the cleaning and disinfection procedures as well as the compliance of the hydrotherapy staff regarding hand hygiene and carriage of PPE. To strengthen and guarantee an accurate surface cleansing and disinfection process after patient usage, a compulsory and easy-to-follow standard operation procedure was developed and implemented. The chemical agents used for disinfection were not changed. Environmental sampling after implementation of the procedure showed no further MRSA contamination. Moreover, a regular (quarterly) environmental sampling after restart of the bathing was conducted to monitor ongoing compliance to the enhanced cleaning and disinfection.

### 3.4. Literature Overview

Our search identified 9 reports that link MRSA outbreaks to hydrotherapy (Table 2). Positive environmental samples were found in 5 reports. Discontinuation of bathing was a frequently reported measure for outbreak control. Moreover, cleaning and disinfection procedures were enforced to address surface contamination.

## 4. Discussion

Contamination of hydrotherapy equipment and hydrotherapy installations in hospitals with MRSA is described in the literature [4]. Especially burn units seem to be at risk for pronounced environmental MRSA contamination [10]. Moreover, MRSA contamination of bathing installations is not limited to the hospital setting but may also be found in the community, such as in athletic training rooms [11]. Acquisition of MRSA from the environment is possible [12] and may play a role for the transmission in nosocomial outbreaks [6]. In the outbreak, we describe growth of the outbreak strain was detected inside and near the bathtub. It remains unclear whether the source for MRSA was the first outbreak patient or an unknown patient who was not tested for MRSA during their clinical stay. The import of MRSA at an earlier time point might have occurred, especially as we found another MRSA isolate in the sink of the tub which was not identical to the outbreak strain. The MRSA outbreak strain may have persisted or may have been repeatedly reintroduced to the bathtub despite disinfection and water change after each patient usage. This assumption seems likely, as the outbreak cases had repeated overlaps in the hydrotherapy unit explaining ongoing transmissions. After the end of the outbreak with an improvement of the cleaning procedure, we restarted hydrotherapy treatment and continued to allow MRSA-positive patients to receive carbon dioxide treatment in the bathtub. According to the principles of infection control, it is crucial to apply such disinfection procedures after each treatment which are able to eliminate all relevant bacteria (whether multidrug resistant or not) in the bathtub in order to prevent cross transmissions between patients. In addition, alternative forms of carbon dioxide application (in particular transcutaneous gaseous application for the extremities [13]) with single-use products may be considered for clinical use.

The additional finding of methicillin-susceptible *Staphylococcus aureus* and *Enterobacter* species in environmental specimens emphasizes the need for optimizing the cleaning and disinfection procedures. Thus, we developed an easy to follow and strict cleaning protocol combined with repeated infection control audits. We did not change the chemical agents used for disinfection as the substances used were bactericidal. We assumed that the contamination was rather caused by an unfavorable work process. The ad hoc discontinuation of the hydrotherapy application after the environmental samples became positive for MRSA led to a rapid outbreak cessation as in other previously reported outbreaks [14–16].

During the outbreak, we discussed a potential (long-term or transient) carriage among the hydrotherapy staff members as a source of MRSA. Nevertheless, we did not perform staff screening but rather enforced usage of PPE and hand hygiene to address potential carriage among the staff.

It is interesting to note that the investigation of a community outbreak of MRSA among professional football players [17] revealed methicillin-susceptible *Staphylococcus* aureus in water samples (taken at the end of a day in a whirlpool) which were identical to those found in nasal swabs. We did not consider the water as a reservoir during our outbreak as there was an exchange after each patient. Nonetheless, we took fresh water samples directly from the pipe system (tap water and carbon dioxide-enriched water) after cessation of the outbreak, and these showed no growth of *Staphylococcus aureus*.

The detection of the outbreak and the identification of the hydrotherapy bathtub as the source for transmission were challenging in the present case. Two of the 3 affected patients of the first outbreak week were treated on the same surgical ward. Thus, we initially suspected on-ward transmission due to transiently contaminated hands of the medical staff in these two patients. We initially did not link the third outbreak case from the tertiary burn unit with the preceding two cases. However, the third case finally was the key to identify the source of the outbreak. This case triggered a deeper analysis of patient movement and treatment data to identify commonalities. The hydrotherapy unit was common to these patients. After the implementation of several infection control interventions including universal decolonization on the tertiary burn unit in the last years, nosocomial MRSA acquisition was practically zero [18], which directed our attention to a potential source outside of the ward. Moreover, this conjecture was supported by the environmental sampling which was very helpful in this outbreak.

Our study has some limitations. First, it is a single-center report and our findings may therefore not be transferable to other hydrotherapy facilities. However, we think that the outbreak experiences reported here may be beneficial for infection control in other institutions. Secondly, due to restricted patient information, we could not evaluate in detail the outcomes due to MRSA in the outbreak patients as suggested by the ORION statement [8].

## 5. Conclusions

Although several infection control measures were implemented in the last 2 decades, MRSA still is a relevant infection control challenge. Best clinical and infection control practices are needed to prevent nosocomial acquisition and outbreaks. The outbreak reported here and other outbreaks link MRSA cross-transmission in the hospital and community settings to hydrotherapy and other bathing facilities. It was demonstrated that MRSA can contaminate the surfaces in bathing facilities. These may serve as an environmental reservoir. This underscores the need for clear concepts regarding surface cleaning and disinfection in order to guarantee high adherence levels. Hospitals should be aware of potential high risk settings for MRSA cross-transmission and it seems helpful to implement regular environmental sampling in these areas to monitor the success of cleaning and disinfection efforts. If patients on different wards acquire nosocomial MRSA, a thorough search for a common source outside of the wards needs to be performed.

## Figures and Tables

**Table 1:** Outbreak cases.

Case	Ward	Underlying disease	Positive MRSA sample in outbreak week	Sites of MRSA isolation	MRSA infection/colonization	Nosocomial MRSA acquisition	MRSA acquisition after begin of carbon dioxide therapy	Outcome
1	Surgical	Burn injury	1	Skin/soft tissue	Colonization	Yes	Within 2 weeks	Discharged from hospital
2	Surgical	Necrotizing fasciitis	1	Skin/soft tissue	Colonization	Yes	Within 2 weeks	Discharged from hospital
3	Burn unit	Electrical injury	1	Skin/soft tissue	Colonization	Yes	Within 2 weeks	Discharged from hospital
4	Internal medicine	Generalized erythroderma	4	Nasopharyngeal, rectal and others not specified	Colonization	Yes	Within 3–4 weeks	Discharged from hospital

**Table 2:** Literature overview of MRSA outbreaks linked to hydrotherapy or other bathing activities.

Report	Persons affected and clinical characteristics	Setting	Role of hydrotherapy/bathing^*∗*^	Control measure regarding hydrotherapy/bathing
1 [19]	9 patients, 6 patients with soft tissue infections, one patient died.	Single-hospital outbreak	MRSA-positive environmental samples from the hydrotherapy department; discussed by the authors as an unclear (secondary) contributor to spread.	Enforcing cleaning in the hydrotherapy department

2 [20]	At least 4 patients, all with clinically relevant infections (bloodstream, respiratory and wound infection).	Single-hospital outbreak	MRSA-positive environmental samples from the hydrotherapy room on the burn unit; discussed by the authors as a potential contributor to spread.	Routine cleaning in the hydrotherapy room

3 [21]	82 mothers with MRSA from episiotomy wound/perineum (46), vaginal discharge (23), urinary tract (9), abdominal wound (2), and breast abscess (2). 28 newborns, 8 newborns had a MRSA conjunctivitis, and the others were colonized.	Single-hospital outbreak	MRSA-positive environmental samples from baths and bidets on a postnatal ward; discussed by the authors as a relevant contributor to spread among other factors such as contaminated mattresses.	Cleaning and refurbishment of baths

4 [15]	37 mothers, 18 newborns, and 9 staff members. Clinically relevant infections occurred in 10 mothers (5 Caesarian section wound infections) and 2 newborns. Most mothers were nasal and perineal carriers of MRSA (23 and 28, respectively). Most newborns were nasal carriers (14) and had positive samples from the umbilicus (9). The staff members were colonized (nasal).	Single-hospital outbreak	MRSA-positive environmental samples from a bath and bidet on an antenatal and neonatal ward; discussed by the authors as a relevant contributor to spread among others.	Discontinuation of bath usage

5 [14]	4 patients with bloodstream infections, one patient died. 1 nurse was colonized (nasal sample).	Single-hospital outbreak	All patients used the same bathtub; environmental samples of the bathtub were not taken; bathtub discussed by the authors as the major contributor to spread.	Discontinuation of bath usage

6 [6]	12 patients. One patient had a skin graft infection. The other patients were colonized (mostly skin and soft tissue).	Multihospital outbreak	MRSA-positive environmental samples from hydrotherapy equipment (stretcher and shower hand held) on a plastic surgery/burn unit; discussed by the authors as the major contributor to spread.	Change of hydrotherapy procedure (no more use of stretchers)

7 [22]	10 football players with skin/soft tissue infections.	Players of a college football team	Usage of whirlpools was discussed by the authors as a potential contributor to spread due to limited water disinfection. However, direct contact during training sessions was presumed to be the most relevant transmission pathway among the players. Water and environmental samples were not taken.	Change of whirlpool practice (water change and disinfection after each usage)

8 [17]	5 football players with skin/soft tissue infections.	Players of a professional football team	Although MRSA was not found in environmental samples, the authors discussed that whirlpool usage might be a potential contributor to spread as methicillin-susceptible *Staphylococcus aureus* strains were found in water samples and in player samples.	

9 [16]	6 neonates with skin/soft tissue infections.	Single-hospital outbreak	Although MRSA was not found in environmental samples, the authors discussed that the discontinuation of bathtub usage was relevant for outbreak control.	Discontinuation of bath usage and implementation of a chlorhexidine-based decolonization for patients

^*∗*^The role of hydrotherapy in each outbreak was categorized as follows: (i) unclear; (ii) potential; (iii) relevant; and (iv) major.

## Data Availability

The data used to support the findings of this study are included within the article.

## Abbreviations

MRSA: Methicillin-resistant *Staphylococcus aureus*
PFGE: Pulsed-field gel electrophoresis
PPE: Personal protective equipment.

## Additional Points

Surface contamination of a hydrotherapy bathtub caused a MRSA outbreak. Environmental sampling was crucial for identifying the outbreak source. Regular environmental samplings can be beneficial in high-risk settings.
